# Aspects of zone-like identity and holotomographic tracking of human stem cell-derived liver sinusoidal endothelial cells

**DOI:** 10.3389/fcell.2025.1528991

**Published:** 2025-10-17

**Authors:** Mikel Amirola-Martinez, Thomas Combriat, Katharina Ferencevic, Ingrid Wilhelmsen, Andrea Dalmao-Fernandez, Petter Angell Olsen, Justyna Stokowiec, Aleksandra Aizenshtadt, Stefan Krauss

**Affiliations:** ^1^ Hybrid Technology Hub, Institute of Basic Medical Research, University of Oslo, Oslo, Norway; ^2^ Department of Immunology and Transfusion Medicine, Oslo University Hospital, Oslo, Norway; ^3^ Njord Center, Department of Physics, University of Oslo, Oslo, Norway; ^4^ Section for Pharmacology and Pharmaceutical Biosciences, Department of Pharmacy, University of Oslo, Oslo, Norway

**Keywords:** LSEC, fenestration, holotomography (HT), pluripotent stem cells (PSC), liver zonation

## Abstract

**Introduction:**

Liver sinusoidal endothelial cells (LSEC) are specialized endothelial cells with unique metabolic and barrier functions adapted to the needs of the liver sinusoid. LSECs are highly sensitive to their environment, and this fragile nature causes challenges in analyzing their phenotype under *in vitro* conditions.

**Methods:**

In this work, we first differentiated LSEC-like cells (scLSECs) from two human pluripotent stem cell lines and characterized them by a panel of qPCR markers, immunohistochemistry, substrate oxidation for energy metabolism, scavenger function, and nitric oxide secretion. We then introduced holotomography, a technique that allows to recover quantitative and three-dimensional information about the refractive indexes of cell components, as a tool to image and track scLSEC *in vitro* in a minimally intrusive, label-free manner.

**Results and discussion:**

Holotomography and developed machine learning-based algorithm for image processing allowed us to describe and monitor changes in intracellular pore-like structures over time. Finally, we tested the possibility of inducing aspects of zone-specific LSEC phenotype and metabolism using culture-based treatments, which resulted in modest shifts in marker expression and metabolic activity. The presented strategy provides an advanced tool kit for investigating liver endothelial cells.

## 1 Introduction

Liver sinusoidal endothelial cells (LSEC) are highly specialized endothelial cells in liver-specific discontinuous vessels called hepatic sinusoids Poisson et al. (2017). Supported by hepatic stellate cells (HSCs), LSECs form the interface between the blood circulation and hepatocytes.

LSECs are essential for the functioning of the liver sinusoid performing a central barrier function while being involved in liver metabolism, synthesis of growth factors, coagulation factor VIII, clearance of pathological agents and scavenging biologic material from the bloodstream, immunological responses and antigen-presenting functions, and architectural maintenance of the liver Sørensen et al. (2012); Bhandari et al. (2021); Fomin et al. (2013). LSECs play a significant role in various liver diseases Bhandari et al. (2021); Poisson et al. (2017), including disease conditions that involve angiogenesis and vasoconstriction. At the same time, LSEC are also involved in liver regeneration following acute liver injury or partial hepatectomy Poisson et al. (2017); McConnell et al. (2023). Furthermore, antigen presentation by LSECs and the secretion of signaling molecules such as nitric oxide (NO) are important factors in ensuring the quiescence of tissue-resident immune cells and hepatic stellate cells (HSCs). Hence, LSECs keep the liver sinusoid in a tight balance, protecting the fragile hepatocytes while tolerating physiological environmental variations Limmer and Knolle (2001); Winkler et al. (2021) and also being major drivers in the onset of and recovery from liver diseases Winkler et al. (2021).

In the healthy adult liver, LSECs are characterized by the formation of transcellular plasmatic discontinuities called fenestrae Poisson et al. (2017); Szafranska et al. (2021). Fenestration, which forms passages through the cytoplasm of LSECs with a diameter ranging between 50 and 300 nm, allows the transport of solutes through the endothelial layer while keeping the blood inside the vessel Szafranska et al. (2021). This structural specialization is crucial for allowing hepatocytes to access circulating nutrients and signaling molecules, while remaining shielded from the mechanical stress and immunological components of the bloodstream Poisson et al. (2017). Fenestrae also allow a fraction of the blood plasma to exit the vessels into the interstitial space called the space of Disse. This controlled leakage slows down the flow speed and facilitates the uptake and processing of molecules such as nutrients and endogenous and exogenous bioactive molecules MacPhee et al. (1995); Chen et al. (2022). The fenestrae are organized in groups called sieve plates, which are formed by tens of fenestrae, with their number and distribution influenced by physiological and pathological conditions Svistounov et al. (2012); Xie et al. (2010). Fenestration and other membrane structures, like transendothelial channels (TEC) can be visualized by several methods, however, at current scanning electron microscopy (SEM) and transmission electron microscopy (TEM) remain the gold standard. SEM visualizes the cell surface with extremely high resolution but requires fixed samples and careful sample preparation involving numerous critical steps. This represents a significant challenge for visualizing and monitoring dynamic changes in structures. Other suitable alternatives such as 3D structured illumination microscopy (SIM) and direct stochastic optical reconstruction microscopy (dSTORM) have been proposed as more accessible optical techniques Mönkemöller et al. (2014); Svistounov et al. (2012). dSTORM relies on specific fluorophores to emit twitching fluorescence, highly improving the resolution in an optical microscopy setup. Although the technique offers enough resolution to image nanoscopic features such as fenestrae and TECs, it still requires membrane staining and special buffers for cell imaging. Another method, photoactivated localization microscopy (PALM), can be performed on live cells but requires endogenous expression of fluorescent proteins, and therefore, cells must be genetically modified before target structures can be imaged Betzig et al. (2006). Atomic force microscopy with an with a specialized imaging mode based on fast acquisition of the force versus distance (FD) curves can be also applied for studying nanostructures in membrane of live LSEC *in vitro* Zapotoczny et al. (2017).

Liver cells, including LSEC, display different phenotypes and functionalities depending on their location along the porto-central axis in the liver lobule. This spatial heterogeneity of liver cells is known as metabolic zonation with three main regions or zones: periportal (zone 1) - cells in proximation to the portal triade, pericentral (zone 3) - cells surrounding the central vein, and middle (zone 2). LSECs from different zones exhibit distinct transcriptomic signature, Halpern et al. (2018), differences in energy metabolism Cunningham and Porat-Shliom (2021), as well as a characteristic fenestration. Periportal LSECs have been reported to display a low abundance of fenestrae with higher diameters, aiding in selective filtration and oxygen supply to hepatocytes. These cells are exposed to higher oxygen levels and nutrient concentrations, influencing their metabolic profile. In contrast, pericentral LSECs, have more fenestrae with smaller diameters, optimizing rapid exchange and waste clearance. This region, with lower oxygen and higher metabolic by-products, aligns LSECs towards glycolysis and detoxification Wisse et al. (1985); Xie et al. (2010). Despite recent advances in understanding the molecular heterogeneity of LSECs through spatial and single-cell omics, their functional diversity and impact on liver homeostasis and disease remains underexplored, largely due to the lack of relevant *in vitro* models that replicate the liver’s zonated environment.

The culture of primary LSEC *in vitro* has been challenging due to limitations in the availability of donor material, the low prevalence of LSEC in tissue, as well as rapid loss of the phenotype and functionality specific to LSEC *in vitro*, including fenestration Juin et al. (2013). LSECs generated from human pluripotent stem cells (scLSEC) can represent a promising alternative to primary LSEC Gage et al. (2020); Wilhelmsen et al. (2023). However, to the best of our knowledge, scLSECs in *in vitro* monoculture were not characterized for fenestration or other membrane pore-like structures, or zonated features.

In this work, we differentiated two pluripotent stem cell lines, the human embryonic stem cell line WAe001-A (H1), and the human induced pluripotent stem cell line UCSFi001-A (WTC11), into stem cell-derived liver sinusoid-like cells (scLSEC_1 and scLSEC_2, respectively). We then tracked the cells from the CD34^+^ progenitor stage towards the specification of scLSEC and further into long-term, post-differentiation *in vitro* culture. The scLSEC lines were characterized by a panel of qPCR markers, immunofluorescent imaging, NO secretion, scavenger function, and substrate oxidation to measure energy metabolism. We then introduced holotomography (HT) as a non-invasive way to track the cell morphology over time, focusing on subcellular structures. HT relies on measuring the different refractive indexes of optic pixels by using the interference between a light path that went through the sample and a reference, allowing to recover the total refractive index along the light path. Doing this at different illumination angles allows reconstructing tomographic stacks containing quantitative refractive index information with 200 nm and 1 µm resolution in the X/Y and Z-axis respectively for the particular setup used in this study Kim et al. (2021). The technique, combined with an in-house developed machine-learning-based algorithm, makes it possible to identify specific cellular structures in 3D on the scLSECs, with high resolution and without the need for labeling or fluorescent protein expression in live cell cultures. We subsequently analyzed the development of characteristic circular pore-like structures in the membrane of scLSECs. Finally, we then tested the effects of supplementary factors that impact metabolic zonation in liver parenchymal cells on the cultured scLSECs, whereby we observed a more marked responsiveness in the scLSECs differentiated from WTC11 hiPSC line (scLSEC_2) compared to H1 hESC-derived scLSECs (scLSEC_1) as judged by expression of zonation markers, energy metabolism and changes in the pore-like structures. The study offers a novel methodological framework for future *in vitro* models with scLSECs and primary LSECs.

## 2 Materials and methods

### 2.1 Cell differentiation into scLSEC

Human pluripotent stem cells were routinely cultured on Geltrex-coated plates (Life Technologies), using Essential 8™ Medium (ThermoFisher Scientific). Two days before starting the differentiation, cells with 50–60% confluency were detached with 0.5 mM EDTA for 3 min and re-plated in a 1:10 ratio in E8 medium containing 10 nM ROCK inhibitor Y-27632 (Stem Cell Technologies). The cell culture medium was replaced the next day and changed every 24 h.

Cell differentiation was performed using our previously published protocol Wilhelmsen et al. (2023), which was based on the work by Gage et al.Gage et al. (2020). The differentiation protocol was performed using male pluripotent stem cell lines: human embryonic stem cell (hESC) line WAe001-A (H1, WiCell Research Institute), and the human induced pluripotent stem cell (hiPSC) line UCSFi001-A (WTC11, Corelli Institute for Medical Research). In short, differentiation was initiated by replacing stem cell medium with base medium as described in Table 1 supplemented with 10 ng/mL BMP4 (Peprotech). On day 1 the medium was replaced with medium supplemented with 10 ng/mL BMP4 and 5 ng/mL bFGF (Peprotech). On day 2, the medium was replaced with the same medium as on day 1 with an additional supplement of 3 μ M CHIR (Biotechne). Next, from day 4 till day 8 cells were treated with 30 ng/mL bFGF, 10 ng/mL VEGF-A 165 (Peprotech), and 10 μ M DAPT (R&D systems) with media replacement every 48 h. On day 8, the cells were sorted using a Dynabeads CD34 Positive Isolation Kit (Life Technologies) and a 15 mL falcon tube Dynamag magnet (Life Technologies). The estimated protocol efficiency was 2%. We resuspended the cells in fresh base medium supplemented with 30 ng/mL bFGF and 10 ng/mL VEGF-A and plated them on Nunclon Delta surface plates coated with 2.5% Geltrex. The differentiation and cultivation of the scLSECs were performed under hypoxic conditions (5% CO_2_).

**TABLE 1:** Cell differentiation media composition. This table describes the basal medium composition of the differentiation, expansion, specification, and scLSEC post-differentiation culture media. A base medium with IMDM and STEMPRO 34 was prepared first and other solutes were then added to the prepared base medium.

Ingredient	Final concentration	Cat.no. Manufacturer
IMDM	75:100	Life Technologies
STEMPRO 34	25:100	Life Technologies
Penicillin/Streptomycin	1:100	Life Technologies
ITS-X	1:10000	Life Technologies
Ascorbic acid-2-Phosphate	50 μ g/mL	Sigma Aldrich
Human recombinant Transferrin	150 μ g/mL	Biogen
Glutamax	2 mM	Life Technologies
Alpha-metil-thyoglutarate	50 μ g/mL	Sigma Aldrich

#### 2.1.1 Cell expansion

The cells were cultured for 3 to 4 passages using plates coated with 2.5% Geltrex and in base medium supplemented with 10 ng/mL VEGF-A and 30 ng/mL of bFGF. The cells were passaged in a 1:2 or 1:3 ratio into new coated cell-culture vessels after detachment by Trypsin/EDTA. Cells were then plated in their final vessel, on Geltrex-coated glass cover-slips for scanning electron microscopy (SEM) and optic microscopy, on Geltrex-coated holotomography (HT)-compatible plates for HT, and cell culture-grade polystyrene plates for other downstream applications.

#### 2.1.2 Cell specification

Cells were specified for 5 days by adding 8-Br-CAMP (Biolog, Germany) and SB-431542 (R&D Systems) to the cell culture medium as previously performed by Gage et al. (2020) and as described in our previous publication Wilhelmsen et al. (2023).

#### 2.1.3 Post-differentiation culture modifications

Generated scLSECs were treated with the potentially zonal-inducing culture conditions for 5 days directly post-differentiation. The treatment mimicking the Z1 environment (the Z1 treatment) consisted of 0.1 μ g/mL glucagon (Sigma Aldrich) and 10 nM C-59 (Tocris). The treatment mimicking the Z3 environment (Z3 treatment) consisted of 10 μ M DAPT (Tocris), 20 ng/mL Wnt2 (Sigma Aldrich), and 50 ng/mL R-Spondin 3 (Peprotech).

#### 2.1.4 Culture of primary human endothelial cells

Commercially available human primary liver sinusoid cells (Axol Bioscience, ax3777-1) were cultured according to the manufacturer’s instructions in LSEC expansion media as specified above. Human umbilical vein endothelial cells (Lonza) were cultured according to the manufacturer’s instructions, in Endothelial Cell Growth Medium-2 (Lonza).

#### 2.1.5 Differentiation and culture of human stem cell-derived hepatic stellate cells

Stem cell-derived hepatic stellate cells (scHSCs) were generated following a previously published protocol Wilhelmsen et al. (2024) using WTC11 cell line. In short, hiPSC were seeded as single cells and cultured on plates coated with 1% (v/v) Geltrex (Thermo Fisher Scientific) in E8 media (Thermo Fisher Scientific) supplemented with 10 μ M Rock inhibitor (STEMCELL technologies). The differentiation protocol was initiated with the following medium: DMEM/F-12 medium with Glutamax™ supplement (Thermo Fisher Scientific) with 1% (v/v) MEM Non-Essential Amino Acids Solution (Thermo Fisher Scientific) and 1% (v/v) B-27™ Supplement (Thermo Fisher Scientific) and Activin A at 100 ng/mL (Peprotech), CHIR at 3 μ M (Tocris), and BMP-4 at 20 ng/mL (Peprotech). Next, from day 1 to day 5, the cells were cultured in a basal mesoderm medium: DMEM/F-12 medium with Glutamax™ supplement containing 1% (v/v) MEM Non-Essential Amino Acids Solution, 1% (v/v) B-27™ Supplement, 0.025% Insulin-Transferrin-Selenium (ITS-G), 2.5 μ M Dexamethasone (Merck Sigma-Aldrich) and 100 μ M 2-Phospho-L-ascorbic acid trisodium salt (Merck Sigma-Aldrich), supplemented with 20 ng/mL BMP-4 on day 1, and 20 ng/mL BMP-4, 20 ng/mL FGF-1 (Peprotech), and 20 ng/mL FGF-3 (R&D Systems) on days 2–5. The differentiating cells were passaged on day 5 in a ratio of 1:3. From day 6 to day 12, the cells were grown in a basal HSC medium consisting of DMEM/F-12 medium with Glutamax™ supplement containing 1% (v/v) MEM Non-Essential Amino Acids Solution, 1% (v/v) Fetal Bovine Serum (FBS) (Thermo Fisher Scientific), 0.025% ITS-G, 2.5 μ M Dexamethasone, and 100 μ M 2-Phospho-L-ascorbic acid trisodium salt, supplemented with 20 ng/mL FGF-1, 20 ng/mL FGF-3, 100 μ M Palmitic acid (PA) (Merck Sigma-Aldrich), and 5 μ M Retinol (ROL) (Merck Sigma-Aldrich) on days 6–12.

#### 2.1.6 Scanning electron microscopy (SEM)

Cell samples cultured on Geltrex-coated slides were fixed using a double fixative solution (2X PHEM, 2% glutaraldehyde (Sigma Aldrich), and 8% Paraformaldehyde) diluted 1:1 with cell culture medium for 15 min. The fixed samples were preserved at 4 °C in DPBS-diluted double fixative (1:1) until further preparation. The samples were then dehydrated by washing in serial incremental alcohol dilutions and dried via the critical point drying technique on a BAL-TEC CPD 030 instrument. The dry samples were coated with a sputter coater with a 10 nm platinum layer using a Leica EM ACE200 instrument. SEM images were acquired with either a Zeiss Gemini SEM 300 or Zeiss EVO50 EP. Images were processed and analyzed with the ImageJ/Fiji (Java 8 32-bit) program.

#### 2.1.7 Holotomographic microscopy

The cell cultures were imaged every 24 h with a Tomocube HT-X1 microscope (Tomocube Inc. South Korea) at 37 °C in a 5% CO_2_-controlled atmosphere. The employed settings were 3D HT with a depth of 70 slices per image set. For the measurement on Figures 4, 5, the images were taken at the same coordinates in all the wells in an automatized manner with equal exposure and focal plane values. For the long-term, post-differentiation culture analysis shown in Figure 4, the image acquisition position varied in every sampling point due to cell-culture vessel characteristics. Correlative imaging for the *in situ* analysis of uptake of Low-Density Lipoprotein from Human Plasma, Acetylated, Alexa Fluor™ 488 Conjugate (Alexa Fluor™ 488 AcLDL) (Thermo Fisher Scientific) was performed on live cells using subsequent 3D HT and fluorescent imaging in Tomocube HT-X1 microscope of the same fields of view and with synchronization of Z-slices. For this cells, we incubated with ac-LDL (15  μ g/mL in phenol red-free cell culture media) for 20 min at 37 °C. Cells were then washed three times with phenol red-free cell culture media and immediately imaged.

#### 2.1.8 Image analysis

**FIGURE 1:**
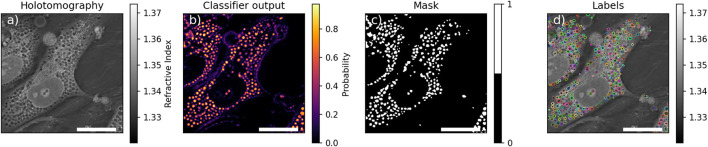
Example of segmentation. **(a)** Holotomographic image, **(b)** probability any given pixel to be part of pore-like structure returned by the classifier, **(c)** resulting mask of the pore-like structure, **(d)** labelled mask after watershed. Scale bar is 10  μm .

**FIGURE 2:**
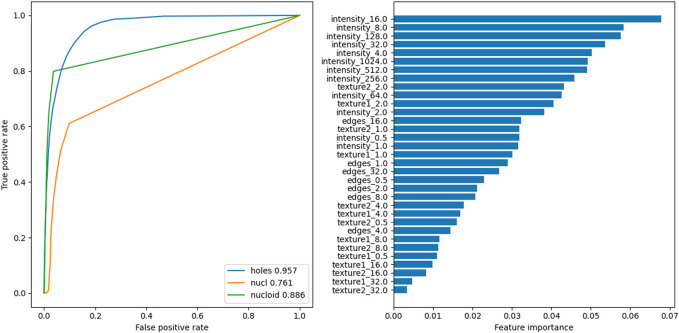
Left: Receiver Operating Characteristic (ROC) curves for the three predictions classes of the Random Forest Classifier on the testing image. Inset: corresponding Area Under the ROC (AUROC). Right: feature importances for the classifier.

The HT image analysis was performed to characterize the observed subcellular circular structures that were classified as pore-like structures (Figures 1a–d). The images were exported and analyzed by a random forest classifier Pedregosa et al. (2011). This classifier was trained on features computed on 6 crops of HT images containing N=737 circular structures, hand-segmented for said circular structures, nuclei, and nucleotids. The features used were computed using the multiscale_basic_features from scikit-imageWalt et al. (2014) with different kernel sizes as summarized in Table 2. The number of estimators (n_est=200) of the classifier was chosen as giving good performances, measured as the Area Under the Curve of the Receiver Operating Characteristic (AUROC) for the circular structures, on a validating dataset composed of one crop containing N=216 structures. The value of the optimal threshold to apply to the probabilities returned by the classifier (see Figures 1b, 2) was chosen to maximize the F-Score of the circular structure detection. Finally, the performances of the algorithm were assessed using a testing dataset of one cropped image comprising N=145 circular structures. The detection of the circular structures and nucleotids on the testing set was very good with an AUROC of more than 0.95 and 0.88, respectively. The performance on the detection of nuclei was more modest (AUROC=0.76) but was deemed sufficient as only an estimation of the number of nuclei present in any given image was sought compared to the full characterization of the circular structure (see after).

**TABLE 2:** Features used for the Random Forest Classifier. Details of implementation can be found in the documentation of multiscale_basic_features from scikit-image Walt et al. (2014). For each feature, kernel sizes were computed from the minimum kernel multiplied by powers of 2 up to the maximum kernel size.

Feature name	Kernel sizes (min - max)
Refractive index (image)	NA
intensity	0.5−1024
texture	0.5−32
edges	0.5−32

To speed up calculations, for any given z-stack, only images where objects were in focus were fed into the classifier. This was done by comparing the mean value of the 2D Laplace operator for each image, $\left\langle \Delta I\right\rangle \left(z\right)$ to the mean of the 2D Laplace operator over the full stack ${\left\langle \left\langle \Delta I\right\rangle \left(z\right)\right\rangle }_{z}$ . Selected images satisfied the criterion (Equation 1):

$$\left\langle \Delta I\right\rangle \left(z_{\text{selected}}\right)>{\left\langle \left\langle \Delta I\right\rangle \left(z\right)\right\rangle }_{z}+n\times \text{std}{\left(\left\langle \Delta I\right\rangle \left(z\right)\right)}_{z},n\in \left\{0,1,2,3\right\}$$ (1)

where n was selected as the highest value which would lead to at least one slice to be analysed. The selected slices were segmented by the classifier and the detected circular structure were individually labeled by a watershed algorithm to separate spuriously connected structures and labels with an eccentricity greater than 0.8, assumed to be false detection, were removed (see Figure 1d). To prevent artificial boosting of the statistics from non-independent images, only the slice containing the highest number of circular structures among the selected slices was kept for further analysis. An example of the final labeled image is shown in Figure 1.

The number of cells in the field of view was estimated by using the masks generated by the classifier for the nucleoli and nuclei. As nucleoli are assumed to be present inside nuclei, the union of these two masks was used and any connected area of more than $185\;\;\;{\text{μm}}^{2}$ was considered a nucleus and incremented the cell count in the field of view by one.

#### 2.1.9 RTqPCR

RNA was isolated using Trizol reagent (Thermo Fisher Scientific) following the standard protocol. cDNA was synthesized from the RNA template using a High Capacity cDNA Reverse Transcription Kit (Thermo Fisher Scientific). RTqPCR was performed on a Viia 7 (Thermo Fisher Scientific) instrument using TaqMan reagent probes (Full list of probes and reagents shown in AppendixSuplementary appendix). TBP was used as housekeeping gene. Relative expression levels of each gene in each sample were determined with ΔΔ Ct method performed by the QuantStudio Real-Time PCR software (Thermo Fisher Scientific).

#### 2.1.10 NO secretion

Nitric Oxide (NO) in the cell culture medium was measured with a Nitric oxide assay kit (Life Technologies) according to the manufacturer’s instructions. The assay standards were prepared in the cell culture medium.

#### 2.1.11 Immunofluorescence and confocal microscopy/structured illumination microscopy (SIM) imaging

Differentiated cells were fixed with 4% paraformaldehyde (Sigma-Aldrich) for 10 min and were then permeabilized and blocked in a permeabilization solution made of 0.1% (v/v) Triton-X (Sigma-Aldrich) and 10% (v/v) Fetal Bovine Serum (Gibco) diluted in DPBS (Gibco) for 10 min. The cells were then incubated overnight at 4 °C with primary antibodies diluted in a blocking solution (10% (v/v) Fetal Bovine Serum (Gibco) diluted in DPBS (Gibco)). Secondary antibody- and nuclear staining with DAPI (Thermo Fisher Scientific) was subsequently performed in the dark for 1 h at room temperature. Primary antibodies used in this study are as follows: anti-LYVE, produced in goat (R&D Systems, 1:50), rabbit anti-LC3B (Abcam, 1:1000), rabbit anti-Caveolin 1 (CellSignaling Technologies, 1:200), mouse anti-Rab7 (CellSignaling Technologies, 1:200), mouse anti-LAMP1 (ThermoFisher Scientific, 1:200). Secondary antibodies are: Alexa Fluor® 488 AffiniPure Donkey Anti-Goat IgG (H + L), Alexa Fluor® 647 AffiniPure Donkey Anti-Mouse IgG (H + L), Cy™3 AffiniPure Donkey Anti-Rabbit IgG (H + L), all from Jackson ImmunoResearch. The glass coverslips with the stained cells were then mounted on glass slides. Confocal imaging was performed using LSM700 (Zeiss, Germany) confocal microscope with standard filter sets, and 63x/1.4 NA oil objective. Structure illumination microscopy (SIM) imaging was performed using the ZEISS Elyra PS1 system with standard filter sets and laser lines with a Plan-APOCHROMAT, 63x/1.4 NA oil objective in the SIM mode of the system. SIM imaging was performed using five grid rotations (0.51 mm grid) for 23 Z-planes with a 0.091 μ m spacing. For SIM image reconstruction, the ZEN black software (MicroImaging, Carl Zeiss) was used with the following “method” parameters: Processing: manual; Noise filter: −5.0; SR frequency weighting: 1; Baseline cut, sectioning: 100/83/83; PSF: theoretical; Output: SR-SIM.

#### 2.1.12 Substrate oxidation assay: cell metabolism

Cell metabolism analysis of both glucose and oleic acid (as a representative free fatty acid) was performed as previously described Wensaas et al. (2007). Cells were plated in a 96-well plate. On the day of the experiment, cell media was replaced either by glucose substrate medium consisting of DPBS supplemented with D-[^14^C(U)]glucose (0.5 μ Ci/mL, 200 μ M), 10 mM HEPES and 10 μ M BSA, or by oleic acid substrate medium prepared in DPBS supplemented with [1–^14^C]oleic acid (0.5 μCi/mL, 100 µM), 10 mM HEPES, 40 µM BSA and 1 mM L-carnitine. A 96-well filter plate (UniFilter® GF/B), activated with 1 M NaOH to capture CO_2_, was placed on top of the cell plate, and the system was incubated for 4 h at 37 °C. Thereafter, cells were washed with PBS and harvested in 0.1 M NaOH. The CO_2_ trapped in the filter and the cell-associated (CA) radioactivity were counted on a 2,450 MicroBeta2 scintillation counter by adding scintillation fluid. All values were then normalized to protein content measured by Bio-Rad protein assay using a VICTOR™ X4 Multilabel Plate Reader (Perkin Elmer). Total substrate uptake and fractional oxidation were calculated from CO_2_ and CA values as: CO_2_+CA and CO_2_/total uptake. Fractional oxidation is a ratio that represents the amount of substrate that has been oxidized based on how much has been uptaken.

#### 2.1.13 AcLDL uptake assay

For flow cytometry-based assay of endocytotic activity, 15  μ g/mL Low-Density Lipoprotein from Human Plasma, Acetylated, Alexa Fluor™ 488 Conjugate (Alexa Fluor™ 488 AcLDL) (Thermo Fisher Scientific) was prepared in 0.5% BSA in DMEM. Cells were detached and incubated in the suspension for 20 min at 37 °C, then washed three times with PBS. Flow cytometry analysis was performed in biological triplicates with a BD Accuri C6 Plus Flow Cytometer and data was analyzed with the native software of the cytometer and histograms were made with Floreada (floreada.io) online software.

#### 2.1.14 Statistical analysis

Independent cell lines and independent differentiation repeats were treated as biological replicates, denoted “n,” and separate wells and independent images within the same cell line and differentiation were treated as technical replicates, denoted “N.” Statistical analysis was performed using GraphPad Prism (version 9–5.0) program. The data was checked for normality using the Shapiro-Wilks test before statistical analysis. The HT data visualized in Figure 4F was cleaned for outliers using the ROUT method with Q = 1% before data visualization and statistical analysis due to the large size of the dataset. The applied statistical analyses are detailed in the figure legends. Statistical significance was determined as p ≤ 0.05 and all statistically significant values are indicated in the figures. When asterisks are used to indicate p-value, they are visualized as follows: ^*^p ≤ 0.05, ^**^p ≤ 0.01, ^***^p ≤ 0.001, and ^***^p ≤ 0.0001.

## 3 Results

### 3.1 Generating and characterizing scLSECs

**FIGURE 3:**
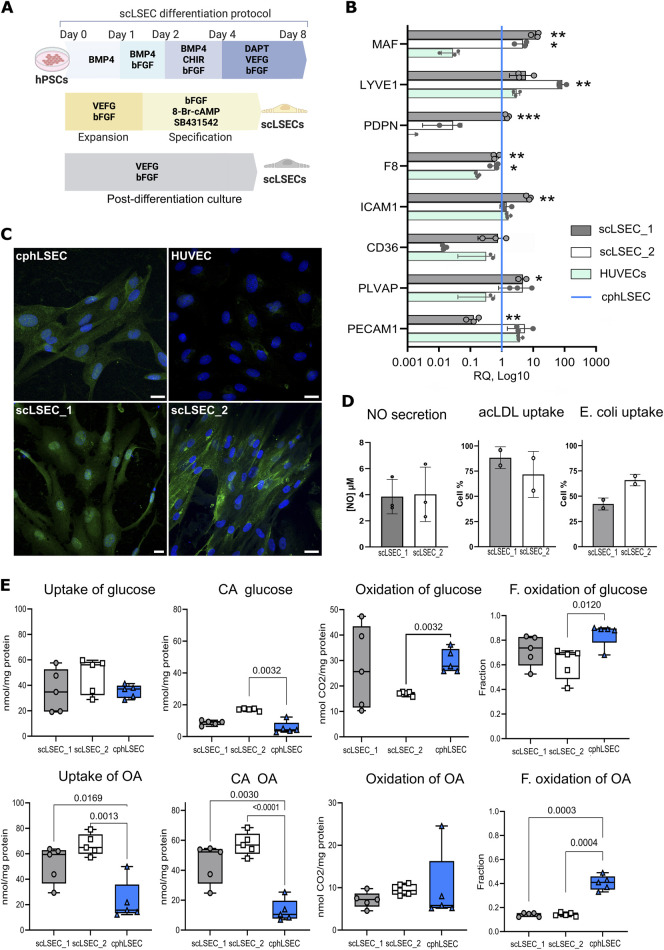
Characterization of stem cell-derived LSECs (scLSEC) as compared to cultured primary human LSECs (cphLSECs). **(A)** Graphical description of the differentiation, specification (yellow), and post-differentiation (grey) scLSEC culture protocol. **(B)** Relative gene expression of LSEC markers in generated scLSEC_1 and scLSEC_2, normalized to *in vitro* cphLSECs (blue line) and compared to HUVECs. The relative expression is presented on a log10 scale. Statistical test: Unpaired t-tests HUVECs, N = 3. **(C)** Representative immunofluorescence images of cphLSEC, HUVECs, scLSEC_1, and scLSEC_2 stained for the LSEC marker LYVE1. Scale bars = 20 μ m. **(D)** Functionality assessment of LSEC_1 and LSEC_2 by nitric oxide (NO) secretion (N = 3) and ligands (acLDL and E.coli bioparticles) binding capacity, as measured by flow cytometry (n = 2 independent differentiations). **(E)** Substrate oxidation assays of glucose and oleic acid (OA) in scLSEC_1, scLSEC_2 and cphLSEC. CA: Cell-associated, F. oxidation: Fractional oxidation. Statistical test: Unpaired t-tests with Welch’s correction compared to cphLSECs. N = 5.

Using a modified version Wilhelmsen et al. (2023) of a published protocol Gage et al. (2020) we generated stem cell-derived liver sinusoidal endothelial cell-like cells (scLSECs), from the human hESC cell line WAe001-A (H1, WiCell Research Institute) (scLSEC_1), and the human hiPSC cell line UCSFi001-A (WTC11, Corelli Institute for Medical Research) (scLSEC_2). A protocol diagram can be seen in Figure 3A. Our previously published article also describes the protocol in detail, including a multi-level characterization of the obtained scLSECs Wilhelmsen et al. (2023).

After 5 days of post-differentiation culture, the two scLSEC lines were compared to commercially available primary human liver sinusoid endothelial cells, suitable for *in vitro* culture (passage 0), and therefore referred to as cultured human primary LSEC (cphLSEC). For broader phenotypic comparison, human umbilical vein endothelial cells (HUVECs) were included as a representation of non-liver endothelial cells. As shown in Figure 3B, generated scLSECs expressed key LSEC transcription factor c-Maf (*MAF*), LSEC marker LYVE1 (lymphatic vessel endothelial hyaluronan receptor 1*, LYVE1*), coagulation factor VIII (*F8*), and plasmalemma vesicle associated protein (*PLVAP*) at the level similar to cphLSEC and higher than in HUVECs. At the same time, CD36 (*CD36*) and podoplanin (*PDPN*) were expressed at lower levels in the scLSECs_2. Immunostaining confirmed the presence of LYVE1 in both scLSECs and cphLSEC, but not in HUVECs, as demonstrated in Figure 3C.

To evaluate the functionality of generated cells, we analyzed nitric oxide (NO) secretion and scavenging capacity of scLSECs as demonstrated in Figure 3D. NO is a vasodilator that regulates tissue homeostasis and is produced and secreted by endothelial cells, preventing sinusoidal capillarization by keeping HSCs quiescent Große-Segerath and Lammert (2021). The NO secretion assay showed no difference between the scLSEC lines. The scavenging function of scLSECs was demonstrated by the endocytic uptake of fluorochrome-labeled acetylated low-density lipoprotein (AcLDL), as well as binding of bacterial (*E.coli*) bioparticles using flow cytometry. Due to limitations in cell number, cphLSECs were not used in these functional assays. Scanning electron microscopy (SEM) demonstrated the presence of scattered pore-like structures (marked with white arrows) on both cphLSECs and scLSECs, which, however, were not clustered in sieve plates and therefore cannot be classified as fenestrae (Supplementary Figure 1A). While the applied SEM preparation protocol lacked postfixation steps that would better preserve membrane integrity and may have introduced minor artefactual depressions, the consistent localization and appearance of these structures suggest that they reflect the membrane structure of scLSEC and cphLSEC (Supplementary Figure S1A).

Lastly, we applied a radioactive substrate oxidation assay to characterize energy metabolism in the scLSECs and cphLSECs. Fatty acids and glucose are primary energy sources, but they follow different pathways for ATP production. Glucose is metabolized via glycolysis to pyruvate that can be further metabolized to acetyl Co-A which enters the Krebs cycle in the mitochondria for ATP production. In contrast, fatty acids like oleic acid (OA) undergo beta-oxidation to produce acetyl Co-A, a energy-intensive process compared to glycolysis. We exposed cells to radio-labeled forms of both glucose and oleic acid, and measured the presence of radioactivity both in the cells and CO_2_ released by cells. The results showed differences between scLSECs and cphLSECs, but also between the two scLSEC lines as seen in Figure 3F. While glucose metabolism was similar across cell types, except scLSEC_2 exhibiting lower glucose oxidation, scLSECs had significantly higher OA uptake and storage but lower OA oxidation compared to cphLSECs, indicating reduced reliance on OA for energy production. In summary, scLSECs differentiated with the current protocol show comparable glucose metabolism to cphLSECs but lower OA oxidation.

In summary, the scLSECs were comparable to cphLSECs by expression of LSEC markers both at the transcriptomic and protein level and displayed LSEC-characteristic functions. However, the scLSECs were less efficient than cphLSECs at utilizing OA as an energy source, highlighting the immaturity of the stem cell-derived scLSEC material.

### 3.2 Holotomography of subcellular structures in scLSECs

LSECs *in vivo* exhibit a unique discontinuous phenotype characterized by transcellular fenestrae organized in sieve plates. This phenotype enables efficient exchange of nutrients, hormones, lipoproteins, and waste products between the blood and hepatocytes, supporting liver metabolism, detoxification, and immune surveillance Szafranska et al. (2021). In addition, there is emerging evidence that transendothelial channels (TECs) or similar transcellular pathways may exist in LSECs in specific contexts (CITATION). Detailed structural analysis of fenestrae can be performed by SEM imaging in fixed and dehydrated samples, which, however, requires long and tedious sample preparation and is not generally suitable to monitor dynamic changes Zapotoczny et al. (2019). Super-resolution optical microscopy with membrane staining enables potential tracking of dynamic changes, but requires specific membrane markers. As an alternative, atomic force microscopy has been applied for the monitoring of the formation or closing of fenestrae in murine LSECs Zapotoczny et al. (2017). Building on initial observation of pore-like structures in scLSEC membranes obtained using SEM (Supplementary Figure S1), we further explored holotomography (HT) imaging as a label-free, live-cell method to expand the toolkit for assessing LSECs morphology and ultrastructure.

**FIGURE 4:**
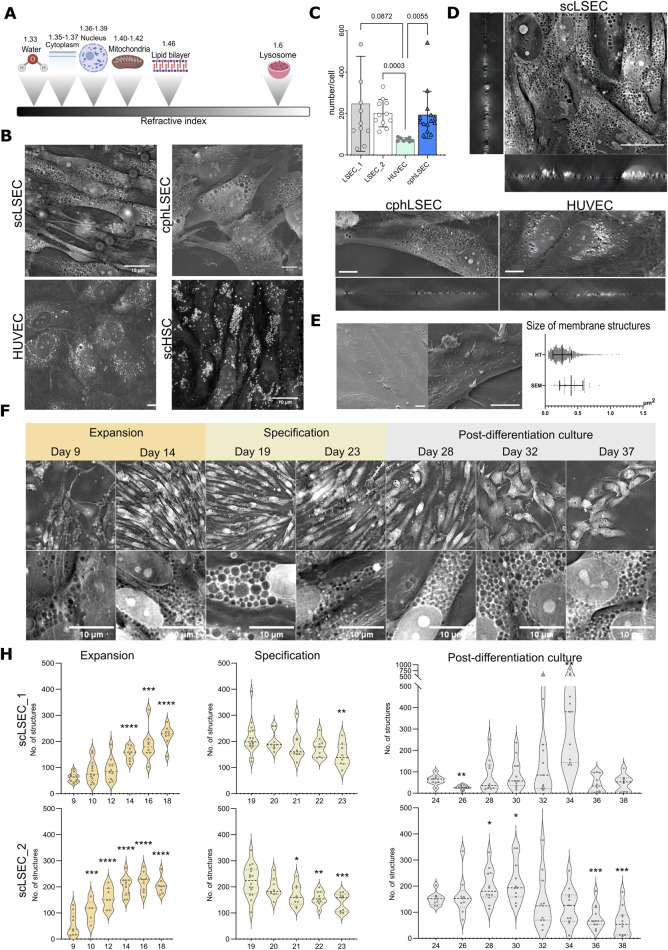
Holotomography (HT) imaging of scLSECs during expansion, specification and post-differentiation culture. **(A)** Scheme showing refractive indexes of cellular components. **(B)** Representative HT images of scLSEC, cphLSEC, HUVEC and stem cell-derived hepatic stellate cells (scHSCs) to visualize and differentiate cell type-specific features. Scale bar = 10 µm. **(C)** Number of pore-like structures in scLSECs, cphLSEC and HUVECs. Statistical test: one-way ANOVA test, N = 8–14 images. **(D)** Orthogonal view of Z-stacks of scLSEC, cphLSEC and HUVECs, demonstrates the presence of pore-like structures spanning through the whole thickness of cells. **(E)** Representative scanning electron microscopy (SEM) images of scLSEC (left) and cphLSEC (right). Scale bars = 10 μ m. Measured area (in μ ${\text{m}}^{2}$ ) of pore-like structures in scLSEC_1 using HT and SEM, showing a partial overlap of the their size. **(F)** Representative HT images and detailed crops of scLSEC_2 from day 9–37 of culture showing an increase during the expansion protocol, followed by a gradual reduction of numbers in the specification phase. Scale bars = 10 µm. **(H)** Changes in the number of circular structures per cell in HT images of scLSECs. N = 5–16 images. Statistical test: Unpaired t-test with Welch’s correction, comparing the first day of each graph to the subsequent days. ^*^p ≤ 0.05, ^**^p ≤ 0.01, ^***^p ≤ 0.001, ^****^p ≤ 0.0001.

HT, an interferometric technique, is a novel approach that measures the refractive index (RI), an intrinsic optical parameter describing the speed of light passing a specific material, to visualize living cells and tissues. As demonstrated in Figure 4A, this method relies on the distinct refractive index of cell compounds and structures to generate an image. HT offers a rapid and non-invasive means of visualizing subcellular structures in living cells at high resolution over extended periods.

Our initial step was to identify distinct structures in scLSECs and compare their HT images with those of other endothelial cells in this study (cphLSECs and HUVECs) and with scHSCs as a non-endothelial reference 4B. As expected, scHSCs displayed numerous lipid droplets with high RI, consistent with previous reports Wilhelmsen et al. (2024), unlike scLSEC and cphLSEC 4B. In contrast, the scLSECs and cphLSEC exhibited abundant circular structures with a RI lower than in the cytoplasm and comparable to background RI values (“low-RI” structures), suggesting that they can represent pore-like structures. HUVECs displayed structures with both high RI and RI at the level of background (low-RI). However, using a machine-learning algorithm for HT images analysis, detailed in the methodology section, we demonstrated that the number of circular structures with background-level RI was significantly lower in HUVECs than in scLSEC and cphLSEC.

Orthogonal reconstructions of Z-stacks from HT imaging demonstrated that a significant part of these low-RI regions spans the whole thickness of cells, as particularly seen in scLSECs and cphLSECs (Figure 4D). The described low-RI structures were similar in size and placement to those visualized by SEM (Figure 4C). Their morphology and size resembled the depressions seen in SEM images (Figure 4D), although a direct comparison revealed differences in area, likely reflecting sample preparation for SEM and methodological differences in image analysis.

To test whether these low-RI structures corresponded to pores or endocytic vesicles, we performed correlative 3D HT and live-cell fluorescence imaging with Alexa-488-labeled acetylated LDL (acLDL) *in situ*. This approach demonstrated that the acLDL signal mostly overlapped with regions with high RI (vesicles, e.g., endosomes or lysosomes), as visualized by HT as bright dots (Supplementary Figure S2A), but not with low-RI structures in question. In addition, markers of endocytic or autophagic pathways, including LC3B (autophagosomes), Rab7 (endosomes), and LAMP1 (lysosomes), did not show high abundance or localization (Supplementary Figures S1C, S2B) as for low-RI structures. These results argue against the interpretation that the low-RI structures can represent intracellular vesicles. In contrast, high-RI structures in HUVECs had a similar pattern as LAMP1-positive vesicles imaged by SIM, confirming that our correlative approach can distinguish vesicular structures.

Taken together, these observations support the description of the low-RI structures as “pore-like”. While, the described pore-like structures did not cluster in groups, and therefore can not be classified as *bona fide* fenestrae of mature LSECs *in vivo*, the high abundance of such pore-like structures could correspond with the presence of transendothelial channels (TECs) in a non-continuous phenotype of immature LSECs. This would be further compatible with visualized by the confocal imaging presence of PLVAP (plasmalemma vesicle associated protein, Supplementary Figure S1D) - a key component of diaphragmed transcellular structures, including TEC, caveolae and fenestrae in immature LSECs Auvinen et al. (2019), as well as positive staining for caveolin 1 (Supplementary Figure S2B).

Using HT with the developed image analysis algorithm, we evaluated the changes in the pore-like membrane structures during the scLSEC differentiation protocol at the stage of progenitor cell expansion, scLSEC specification, and post-differentiation scLSEC culture. Representative images of different time points are shown in Figure 4E. The analysis revealed that the number of the pore-like structures per cell increased during the expansion stage, decreased throughout the specification stage, and were generally stable with scLSEC line-dependent sporadic variations in the post-differentiation culture (Figure 4F). This suggests that culture treatments designed to induce the LSEC-like phenotype affects observed pore-like structures in the cells, especially evident in the expansion stage, further suggesting a relationship between the LSEC phenotype and the observed structures. At the same time, the size of pore-like structures wasn’t altered during specification with an increased variability in the post-differentiation culture (Supplementary Figure S1A).

In conclusion, while the identity of the circular structures cannot be determined by HT alone, HT imaging combined with the machine-learning algorithm provides a novel method for continuous tracking of the fine subcellular structures in scLSECs, including pore-like structures in membrane.

### 3.3 Aspects of zonal identity after culture treatments of the scLSECs

**FIGURE 5:**
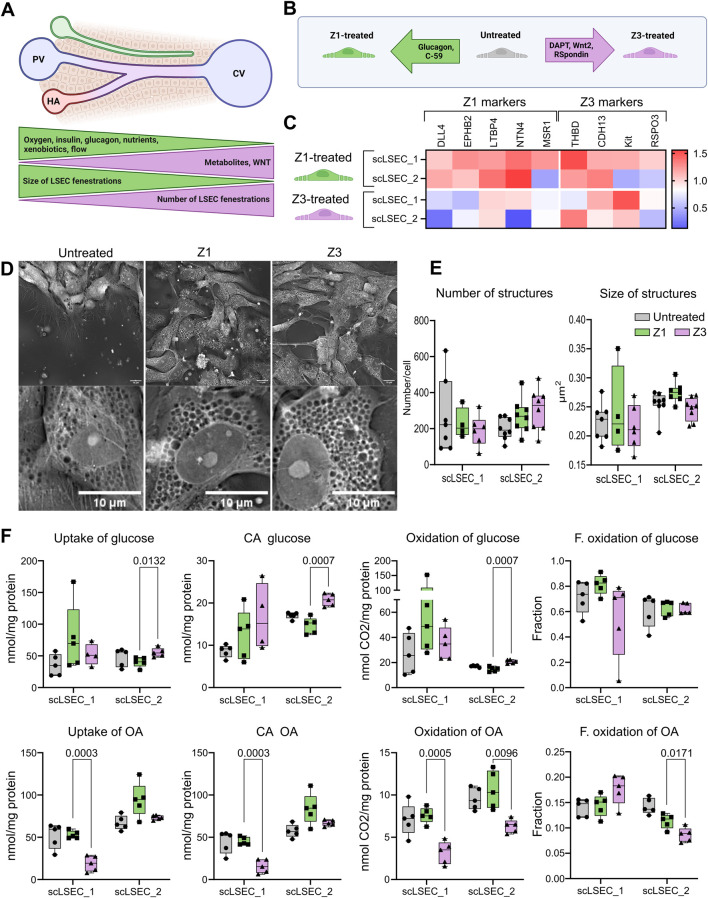
The effects of zonal culture conditions on the scLSECs. **(A)** Representation of a liver sinusoid displaying factors driving metabolic zonation, and zone-specific phenotype of LSEC. PV: portal vein, CV: central vein, HA: hepatic artery. **(B)** Graphic representation of the experimental conditions tested in this experiment. Z1: Zone 1, Z3: Zone 3. **(C)** Heat map of the expression of LSEC markers showing their differential up or downregulation compared to untreated LSECs after 5 days of treatment with Z1 or Z3 inducing cocktails. N = 2-9 replicates from n = 1-3 differentiations per cell line. The relative values are normalized to the untreated condition of each respective cell line. **(D)** Representative holotomography images of scLSEC_2 after 5 days of treatment with Z1 or Z3 inducing cocktails and untreated control. Scale bars = 10 μ m. **(E)** Average number and size of pore-like circular structures detected per cell at day 5 of the treatments. N = 4-8 images. Statistical test: Unpaired t-test with Welch’s correction, comparing Z1 and Z3 for each cell line. **(F)** Metabolic activity assessment for glucose and oleic acid (OA) in untreated control scLSECs, and in scLSECs after 5 days of treatment with Z1 or Z3 inducing cocktails showing zone specific differences in OA uptake and oxidation. CA: Cell-associated, F. oxidation: Fractional oxidation. Statistical test: Unpaired t-test with Welch’s correction, comparing Z1 and Z3 for each cell line. N = 5.

Zonation is a physiological phenomenon of the liver that describes a spatial series of acquired characteristics along the portal-central vein axis (Figure 5A) Droin et al. (2021); Ben-Moshe and Itzkovitz (2019). LSECs are reported to harbor distinct zonal identities in the periportal zone, termed zone 1 (Z1), and in the pericentral zone, termed zone 3 (Z3) Droin et al. (2021); Ben-Moshe and Itzkovitz (2019). Hence, after defining the LSEC phenotype of the scLSECs (Figure 3) and characterizing their pore-like structures by image analysis (Figure 4), we aimed to assess the impact of zone-specific culture conditions on the cells. The sLSECs were treated with media combinations designed to mimic the zonal conditions of Z1 and Z3 for 5 days to attempt an *in vitro* induction of the zonal LSEC identities. A comprehensive set of culture supplements was tested for the induction of zonal scLSEC identities as summarized in Supplementary Table S1, and the most promising conditions are shown in Figure 5. Specifically, glucagon and the Wnt signaling pathway inhibitor C-59 were added to the Z1 media, while the Notch signaling inhibitor DAPT and the Wnt signaling proteins Wnt2 and R-Spondin 3 were added to the Z3 media (Figure 5B). Non-supplemented medium was used as an untreated control.

First, the gene expression of markers known to be modulated in a zone-specific manner was investigated Su et al. (2021). The chosen markers were Delta Like Canonical Notch Ligand 4 (*DLL4*), Ephrin B2 (*EFNB2*), Latent Transforming Growth Factor Beta Binding Protein 4 (*LTBP4*), Netrin 4 (*NTN4*), and Macrophage Scavenger Receptor 1 (*MSR1*) for the Z1-like phenotype and Thrombomodulin (*THBD*), Cadherin 13 (*CDH13*), KIT Proto-Oncogene Receptor Tyrosine Kinase (*KIT*), and R-Spondin 3 (*RSPO3*) for the Z3-like phenotype. Interestingly, the Z1 treatment led to an overall trend of increased Z1 marker expression in both cell lines, while the Z3 treatment generally lowered the expression of the same markers. The tendency of Z3-treated scLSECs to show reduced expression of selected Z1 markers was in particular seen in scLSEC_2 (Figure 5C). Conversely, Z3 markers did not respond to the treatments in a discernible direction. Common LSEC markers were generally unaffected by the treatments (Supplementary Figure S3). Supplementary Figure S2. Next, we evaluated whether discernible morphological changes in the treatment groups could be identified by HT imaging. The morphology of the differentially treated scLSECs was visually similar (Figure 5D) and, we detected no statistically significant differences in the pore-like structures between the Z1-and Z3-treated cells of either scLSEC line. However, a tendency of increased numbers and reduced size (p = 0,08) of pore-like structures in Z3-treated cells when compared to Z1-treated cells was discernible in scLSEC_2, features that would be compatible with a Z3 phenotype (Figure 5E).

Lastly, we investigated changes in glucose and OA metabolism after the treatments, comparing Z1 and Z3 conditions (Figure 5F). The interventions modestly affected energy metabolism in a scLSEC line-dependent manner. A higher uptake and oxidation of glucose was seen in Z3 (compared to Z1) treated scLSEC_2 but not in scLSEC_1, while a reduced uptake and oxidation of OA was visible in Z3 (compared to Z1) of both cell lines. In particular, the scLSEC_2 line showed enhanced glucose uptake reflected in a similar decrease in CA glucose and glucose oxidation. Both scLSEC_1 and scLSEC_2 displayed a pattern of reduced OA uptake, CA OA, and OA oxidation during the Z3 treatment compared to the Z1 treatment. Additionally, the changes in scLSEC_2 OA metabolism were reflected in a decreased fractional OA oxidation, indicating that the OA taken up was oxidized to a lesser extent during Z3 treatment.

In summary, the treatment of scLSECs with media representing zonal interventions led to modest, zone-specific changes, in particular in the scLSEC_2 cell line.

## 4 Discussion

### 4.1 scLSEC as a model of LSEC

Primary human liver sinusoidal endothelial cells (LSECs) are highly specialized endothelial cells, that are limited both by low availability of donor tissue material, challenges in their isolation and preservation of the phenotype in culture. To overcome the limitation of low availability of LSECs, we generated liver sinusoidal-like endothelial cells from human pluripotent stem cells (scLSECs) and characterized their morphology, molecular profile, and selected functional properties. We benchmarked scLSECs against commercially available cultured primary human LSECs (here referred to as cphLSECs) and to non-liver endothelial cells (HUVECs). While cphLSECs are often used as a reference for LSEC identity, they show partial dedifferentiation and do not fully replicate *in vivo* sinusoidal features. This underscores a general challenge in the field and highlights the importance of clearly defining cell types and culture conditions. Particularly, scLSEC displayed high expression of key LSEC markers (Figure 3B) including c-Maf - a critical driver of LSEC identity Gómez-Salinero et al. (2022), expression of the LSEC-typical marker LYVE1 (Figure 3C), and detectable NO secretion (Figure 3E). scLSECs also demonstrated high scavenging capacity, tested by uptake of acLDL and E.coli bioparticles. Importantly, we identified abundant pore-like structures in both scLSECs and cphLSECs. The number of the pore-like structures in these cells was significantly higher than in HUVECs, and was dynamically changed during culture time. Non-endothelial cell type exemplified by scHSC didn’t show the presence of those structures. Both scLSEC lines diverged from the cphLSECs in the metabolism of lipids as measured by the uptake and storage of OA (Figure 3F). Notably, the cphLSECs used in this study more efficiently utilized OA for oxidation, an indicator of mitochondrial fatty acid oxidation and oxidative phosphorylation, characteristic of differentiated cells Shyh-Chang et al. (2013). A possible explanation of the reduced OA oxidation may be the lowered expression of the fatty acid membrane receptor CD36, which is important for the control of fatty acid oxidation when present in the mitochondrial membrane Mallick and Duttaroy (2022). Hence, although the scLSECs are similar to the cphLSECs in several aspects, the scLSECs are still more naive and fetal-like in their phenotype, a limitation that needs to be considered in studies involving hPSC-derived material in general. While the here generated cells exhibited several *bona fide* markers of LSECs, achieving a more complete recapitulation of the adult LSEC phenotype will likely require advancement of *in vitro* culture, for example, 3D culture, application of flow stimulation, refined extracellular matrix (ECM) and co-culture with hepatic stellate cells and hepatocytes. Endothelial cells are known to adapt dynamically to their microenvironment, and this plasticity offers an opportunity for refining scLSEC identity post-differentiation by the modulation of their microenvironment *in vitro*, further promoting maturation and improved functionality of generated cells. For instance, in the original protocol by Gage et al. (2020); Gage et al. (2020) the final maturation, including the acquisition of fenestration, was achieved by transplantation of generated cells into the liver of adult mice. Overall, the robust acquisition of liver endothelial identity at the molecular level, particularly the expression of LSEC-defining transcription factors such as c-Maf, establishes scLSECs as a starting point for building physiologically relevant models of liver sinusoids.

### 4.2 Holotomography can be a useful tool to follow cellular changes in scLSEC

HT microscopy is a powerful tool capable of 3D, live-cell, label-free imaging for studying subcellular structures with a high resolution. Using this tool, we were able to image scLSECs during the entire lifespan of the extensive *in vitro* culture (Figure 4E) in a semi-automated manner, producing a comprehensive, novel dataset. We described subcellular, circular pore-like structures of a similar scale to those observed by SEM imaging on the scLSEC and cphLSEC surfaces (Figure 4D). To analyze those structures we developed a machine-learning algorithm for imaging analysis, characterizing their development throughout the scLSEC differentiation and extended post-differentiation cultivation.

The circular pore-like structures were identifiable by a refractive index lower than the surrounding cytoplasm. Due to the label-free acquisition of the dataset, we cannot confidently determine the identity of the structures at this point, however, correlative imaging with endosomes, autophagosomes and lysosomes markers, as well as *in situ* uptake analysis of acLDL argued against description of them as vesicles, while orthogonal reconstruction of Z-stacks from HT imaging demonstrated that these structures span through the whole thickness of cells and therefore resemble pore-like structures. Observed structures didn’t cluster into sieve plates as it would be typical for fenestra in adult LSEC Szafranska et al. (2021). Additionally, a part of the structures appeared to be only on one side of the cells and therefore can represent caveolae. As such the observed structures may sort under TECs. Importantly, both scLSEC (after specification) and cphLSEC had a significantly higher number of pore-like structures than HUVECs. We acknowledge that further work is needed to determine the precise nature of these structures.

### 4.3 Culture treatments may lead to the development of zone-specific phenotypes

The zonation of non-parenchymal liver cells is a relatively recent discovery Su et al. (2021) and the possibility of *in vitro* induction of zone-specific identities in LSECs is, hence, an understudied topic. Here, we attempted to induce Z1-and Z3-like identities in the scLSECs by treatments with nutrients and signaling molecules characteristic of the respective zones with modestly optimistic results (Figure 5). We observe that the Z1 markers displayed a pattern of general increase during the Z1 treatment, consisting of glucagon and C-59, and decrease during the Z3 treatment, consisting of DAPT, Wnt2, and R-Spondin 3 (Figure 5C), indicating the induction of zonal identities. Additionally, we detected treatment-induced changes to the energy metabolism. The metabolism of glucose can be used to assess glycolysis, a low-energy metabolic process most common in Z3 Cunningham and Porat-Shliom (2021). Correspondingly, we observe an increase in glucose uptake and subsequent CA glucose and glucose oxidation in the Z3-treated scLSEC_2 line. Importantly, the metabolism of OA can be used to assess β -oxidation, a metabolic process prevalent in Z1 Cunningham and Porat-Shliom (2021). The observed trend of higher OA uptake, CA OA, and OA oxidation in both Z1-treated scLSEC lines is encouraging and indicates that the treatments modestly induced zone-dependent metabolic patterns in the scLSECs. Due to the limitation of cphLSEC availability, zonation experiments were performed only on scLSEC.

Although tested culture treatments with zone-mimicking media supplements modestly induced aspects of zonal identities in scLSECs, in particular in scLSEC_2, further work is needed to better recapitulate LSEC zonation features. The limited and non-uniform changes in gene expression and energy metabolism across cell lines suggest that the current approach may not sufficiently emulate the complexity of the liver sinusoidal microenvironment. Given the known plasticity of endothelial cells and their high adaptability to mechanical and biochemical cues, the absence of physiologically relevant gradients of factors, flow stimulation, tissue-like extracellular matrix and/or cell-to-cell interactions may explain the limited zonal responsiveness observed. The transition from simplified static 2D mono-culture of LSEC to multicellular dynamic system can provide a necessary microenviroment for more functional and zone-diverse scLSEC. Such platform can be established by the application of microphysiological systems and can incorporate other liver cells (hepatocytes and hepatic stellate cells), dynamic gradient of soluble factors (e.g., Wnt, nutrients, hormones) and oxygen. Importantly, the extracellular matrix composition, if used for such system, should recapitulate liver specific features, as low stiffness and proteins composition. The impact of each of listed factors can be further evaluated, possibly providing more information on the mechanisms of LSEC zonation. Accordingly, the proposed media composition can be further tested on the scLSEC advanced culture, to test whether enhanced microenvironmental fidelity improves zonal differentiation. Furthermore, additional functional tests, e.g., NO secretion and bioparticle uptake assays can complement molecular profiling and provide more comprehensive assessments of induced zone-specific functionality. This study nevertheless provides a starting point for further examination of the unexplored field of *in vitro* scLSEC zonation.

### 4.4 Conclusion

In this work, we demonstrate that scLSECs are viable alternatives to cphLSECs for *in vitro* LSEC modeling. Furthermore, we identified and characterized circular, pore-like structures by HT imaging by utilizing a machine-learning algorithm, introducing a minimally invasive and non-destructive method for the extended tracking of subcellular structures. Lastly, we demonstrate aspects of zonal identities in the *in vitro*-cultured scLSECs induced by zone-specific media supplements. Collectively, the study demonstrates the utility of scLSECs as *in vitro* LSEC models.

## Data Availability

The raw data supporting the conclusions of this article will be made available by the authors, without undue reservation.
