# Supplementary material for: Aspects of zone-like identity and holotomographic tracking of human stem cell-derived liver sinusoidal endothelial cells
Source: Front Cell Dev Biol. 2025 Oct 17;13:1528991. doi: 10.3389/fcell.2025.1528991 (PMC12575375; doi:10.3389/fcell.2025.1528991)
Supplement: Supplementary file 1 [file DataSheet1.pdf]

Supplementary Material

1 SUPPLEMENTARY TABLES AND FIGURES

Table S1. Conditions tested for the induction of zonal identities in scLSECs. Each row represents one condition tested on at least one scLSEC line. The compounds were added to the scLSEC culture media at the final concentration detailed in parenthesis.

Zone	Compound 1	Compound 2	Compound 3	Compound 4
Z1	Glucagon (1 µg/mL)			
Z1	Glucagon (0.1 µg/mL)			
Z1	Glucagon (0.1 µg/mL)	C-59 (10 nM)		
Z1	Glucagon (0.1 µg/mL)	Normoxia		
Z1	Glucagon (0.1 µg/mL)	Litocholic acid (10 µM)		
Z1	Glucagon (0.1 µg/mL)	Litocholic acid (10 µM)	Vitamin K2 (10 µM)	
Z1	Glucagon (0.1 µg/mL)	Litocholic acid (10 µM)	Vitamin K2 (10 µM)	C-59 (10 nM)
Z1	Normoxia			
Z3	DAPT (10 µM)			
Z3	DAPT (10 µM)	LECT2 (30 ng/mL)		
Z3	DAPT (10 µM)	Wnt2 (20 ng/mL)		
Z3	DAPT (10 µM)	Wnt9b (20 ng/mL)		
Z3	DAPT (10 µM)	CHIR (3 uM)		
Z3	DAPT (10 µM)	R-Spondin 1 (50 ng/mL)		
Z3	DAPT (10 µM)	R-Spondin 3 (50 ng/mL)	Wnt2 (20 ng/mL)	
Z3	IOX2 (30 µM)			
Z3	IOX2 (30 µM)	R-Spondin 1 (50 ng/mL)		
Z3	LECT2 (30 ng/mL)			
Z3	LECT2 (30 ng/mL)	Retinoic acid (0.1 nM)		
Z3	LECT2 (30 ng/mL)	Retinol (0.1 nM)	CHIR (3 µM)	
Z3	LECT2 (30 ng/mL)	Retinol (0.1 nM)	DAPT (10 µM)	
Z3	Retinoic acid (0.1 nM)			
Z3	Wnt2 (20 ng/mL)			
Z3	Wnt9b (20 ng/mL)			

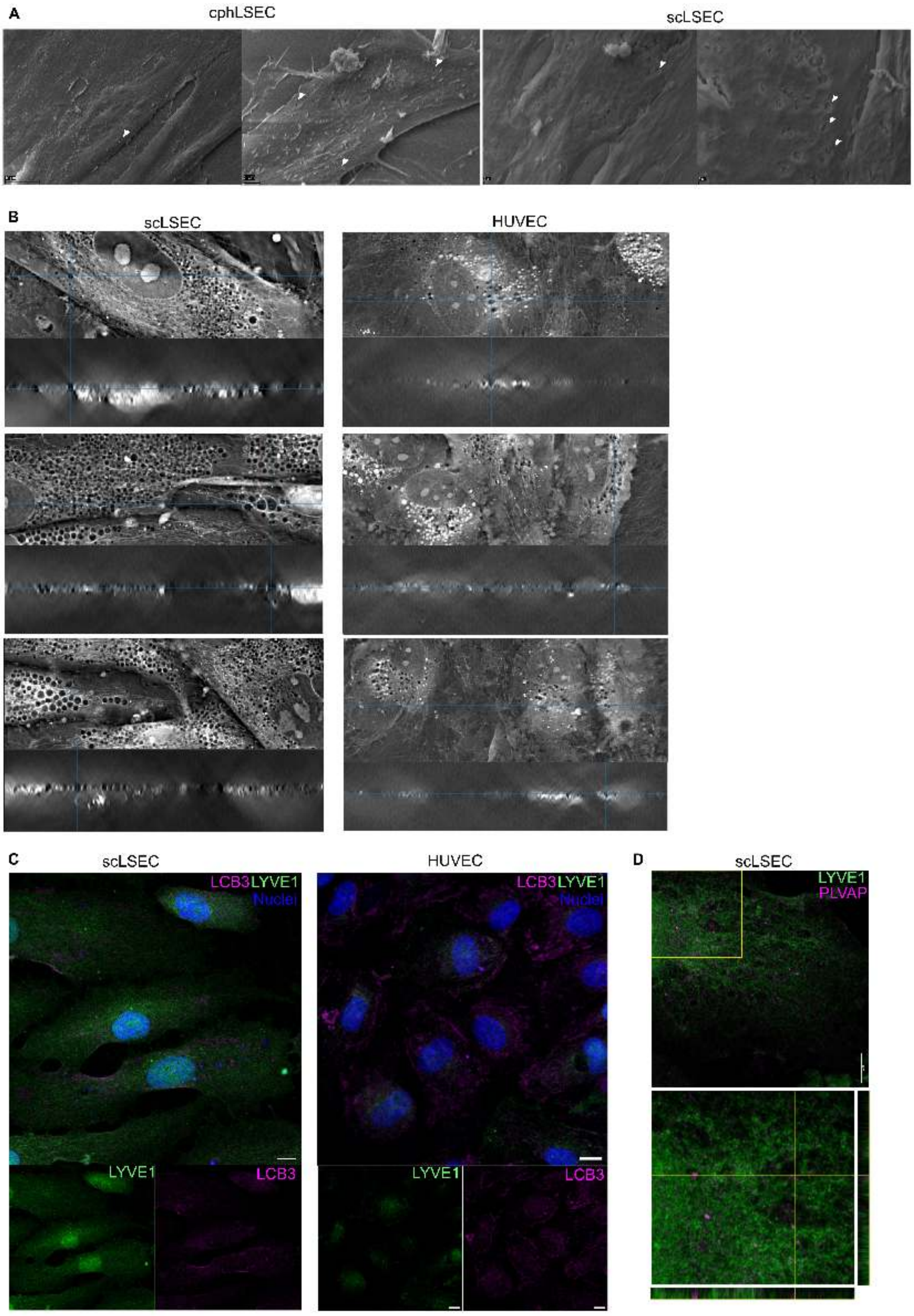

Figure S1. A. Representative scanning electron microscope (SEM) images of cphLSEC and scLSEC 2 demonstrating the presence of membrane depressions. Examples of such structures are highlighted with white arrows. Scale bars = $2\,\mu m$. **B.** Representative HT images of scLSEC and HUVECs with corresponding orthogonal view of Z-stack (70 stacks), demonstrates the presence of pore-like structures spanning through the whole thickness of cells. **C.** Confocal imaging of scLSEC and HUVECs stained with antibodies against the LSEC marker LYVE1 (green) and autophagosomes marker LC3B (magenta). Scale bars = $10\,\mu m$. **D.** Confocal imaging of scLSEC demonstrating presence of PLVAP and absence of its overlap with LYVE1. Scale bar = $10\,\mu m$.

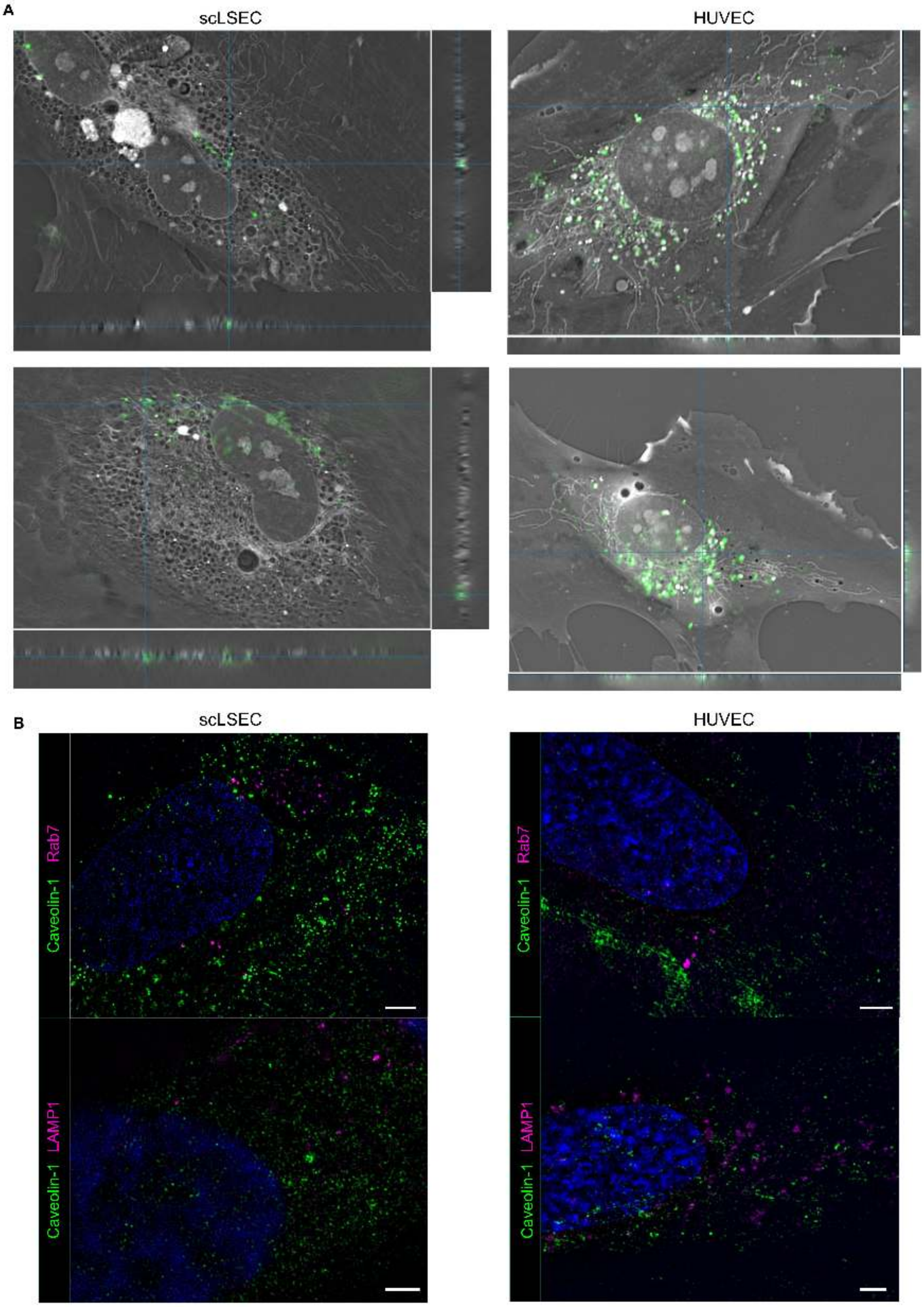

Figure S2. A. Representative images of correlative HT and fluorescent imaging of scLSEC and HUVECs after incubation with fluorochrome-labelled acLDL (green). Corresponding orthogonal view of Z-stack compiled from both HT and fluorescent imaging, demonstrates the presence of pore-like structures spanning through the whole thickness of cells, which however, not labelled with acLDL. **B.** SIM imaging of scLSEC and HUVECs stained with antibodies against caveolin-1 (green), Rab7 and LAMP1 (magenta). Scale bars: $2\,\mu m$.

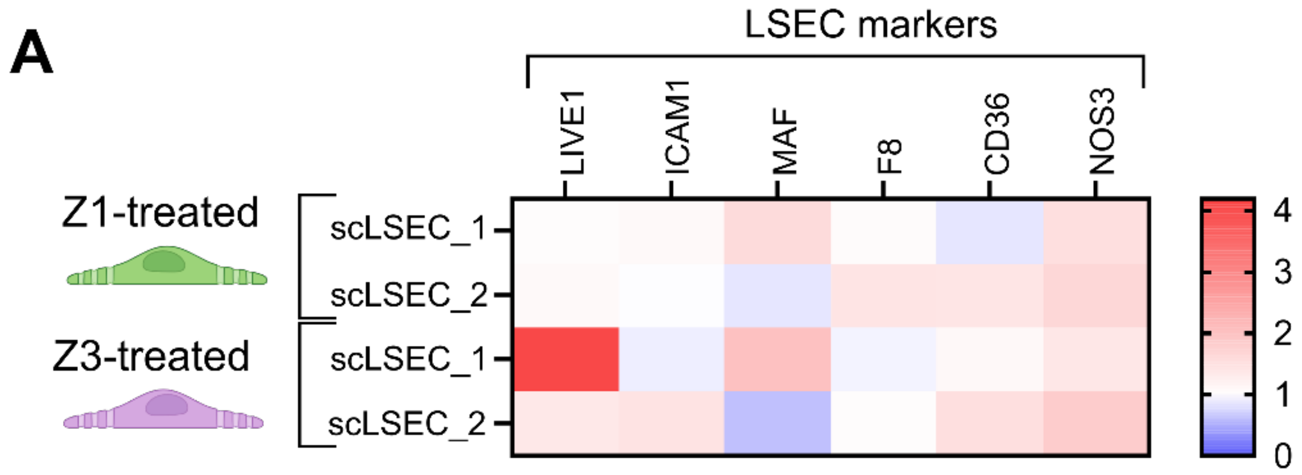

Figure S3. LSEC identity markers after zonal treatment of scLSECs. **A.** Transcriptomic expression of commonly expressed LSEC markers after 5 days of Z1- or Z3 treatment. N = 3-9 replicates from n = 1-3 differentiations per cell line. The relative values are normalized to the untreated condition of each respective cell line.
